# Temperature dependence of the mutation rate towards antibiotic resistance

**DOI:** 10.1093/jacamr/dlae085

**Published:** 2024-06-06

**Authors:** Timo J B Van Eldijk, Eleanor A Sheridan, Guillaume Martin, Franz J Weissing, Oscar P Kuipers, G Sander Van Doorn

**Affiliations:** Groningen Institute for Evolutionary Life Sciences, University of Groningen, Groningen, The Netherlands; Department of Medical Microbiology and Virology, Carl von Ossietzky University Oldenburg, Oldenburg, Germany; Groningen Institute for Evolutionary Life Sciences, University of Groningen, Groningen, The Netherlands; Institut des Sciences de l’Evolution de Montpellier UMR5554, Université de Montpellier, CNRS-IRD-EPHE-UM, Montpellier, France; Groningen Institute for Evolutionary Life Sciences, University of Groningen, Groningen, The Netherlands; Groningen Biomolecular Sciences and Biotechnology Institute, University of Groningen, Groningen, The Netherlands; Groningen Institute for Evolutionary Life Sciences, University of Groningen, Groningen, The Netherlands

## Abstract

**Objectives:**

Environmental conditions can influence mutation rates in bacteria. Fever is a common response to infection that alters the growth conditions of infecting bacteria. Here we examine how a temperature change, such as is associated with fever, affects the mutation rate towards antibiotic resistance.

**Methods:**

We used a fluctuation test to assess the mutation rate towards antibiotic resistance in *Escherichia coli* at two different temperatures: 37°C (normal temperature) and 40°C (fever temperature). We performed this measurement for three different antibiotics with different modes of action: ciprofloxacin, rifampicin and ampicillin.

**Results:**

In all cases, the mutation rate towards antibiotic resistance turned out to be temperature dependent, but in different ways. Fever temperatures led to a reduced mutation rate towards ampicillin resistance and an elevated mutation rate towards ciprofloxacin and rifampicin resistance.

**Conclusions:**

This study shows that the mutation rate towards antibiotic resistance is impacted by a small change in temperature, such as associated with fever. This opens a new avenue to mitigate the emergence of antibiotic resistance by coordinating the choice of an antibiotic with the decision of whether or not to suppress fever when treating a patient. Hence, optimized combinations of antibiotics and fever suppression strategies may be a new weapon in the battle against antibiotic resistance.

## Introduction

The emergence of antibiotic resistance is at its core an evolutionary process, with recent research showing that antibiotic resistance can evolve within a patient within just 3 days.^1^ More specifically, it may be viewed as an ‘evolutionary rescue’ process; when treatment commences, a bacterial population needs to rapidly evolve resistance before it is driven to extinction by antibiotic exposure.^2,3^ The probability that the bacterial population evolves resistance is dependent on the rate of occurrence of mutations that confer resistance. The higher the mutation rate towards resistance, the greater the probability that antibiotic treatment fails due to the emergence of resistance.^2,3^

Mutation rates in bacteria are not constant but are instead dependent on environmental conditions such as temperature.^4,5^ Most biochemical processes increase with temperature, so it has been speculated that mutation rate would be positively associated with temperature.^6^ In addition, bacterial mutation rates can be regulated to become elevated in stressful environments, a phenomenon known as stress-induced mutagenesis.^7^ Pathogenic bacteria may therefore express a different mutation rate when their (human) host responds to an infection with fever—an increase in the body temperature above 38.3°C—which can create mildly stressful conditions for an infecting bacterial population.^8^ This leads to a question that, to our knowledge, has not been addressed before: How does the increase in temperature associated with fever influence the mutation rate towards antibiotic resistance? If mutation rates towards antibiotic resistance are elevated at fever temperatures, fever suppression (using antipyretic drugs such as acetaminophen)^8^ could be a successful tactic to lower the mutation rate and thus combat the emergence of resistance.

Therefore, we examined the temperature dependence of the mutation rate towards antibiotic resistance in *Escherichia coli*. We assessed the mutation rate at two temperatures: 37°C (representing a normal body temperature) and 40°C (representing a typical fever temperature). We used a fluctuation-test approach to determine the mutation rate towards antibiotic resistance for three different antibiotics, each with a different mode of action: ciprofloxacin (a topoisomerase and DNA-gyrase inhibitor), rifampicin (a DNA-dependent RNA polymerase inhibitor) and ampicillin (a peptidoglycan transpeptidase inhibitor).^9–11^

## Methods

We used *Escherichia coli* strain REL4548-CFP-lux, a strain from Lenski’s long-term evolution experiment, transformed by chromosomic insertion of a cyan fluorescent protein (CFP) reporter gene and lux genes (see Supplementary material, available as Supplementary data at *JAC* Online).^12–14^ We estimated the mutation rate towards antibiotic resistance using a fluctuation test.^15,16^ Three experiments were performed for each antibiotic in Groningen; an additional two were conducted for ampicillin in Montpellier (the protocol for these additional experiments deviates slightly; see Supplementary material 1.3). For each experiment and each temperature (37°C and 40°C), 40 replicate populations, all originating from one dilute cell suspension (approximately 100 or 200 cells per population depending on the experiment, see Supplementary material 1.2), were grown in liquid culture. When populations were in the mid-exponential phase, population density and the number of antibiotic-resistant mutants were assessed. Population density was estimated using two methods: counting cfus and measuring the fluorescence of CFP (results shown in Supplementary material 2.4). The number of resistant mutants in each population was assessed by plating the population on an antibiotic-containing agar plate. These antibiotic-containing agar plates were incubated at the same temperature at which the populations were initially grown, i.e. a population grown at 37°C was plated at 37°C. Finally, the R package FLAN was used to infer mutation rates using several different estimation methods (shown in Supplementary material 2.5) and conduct a fluctuation analysis test comparing the mutation rates at the two temperatures.^17^ There was a significant specific culture effect for mutation rates, so the experiments for each antibiotic were analysed in a pairwise manner—i.e. for each comparison, mutation rate estimates were obtained for both temperatures on the same day and from an identical starting culture (except one of the two ampicillin experiments performed in Montpellier).

## Results

### Temperature dependence of the mutation rate

**Figure 1.**
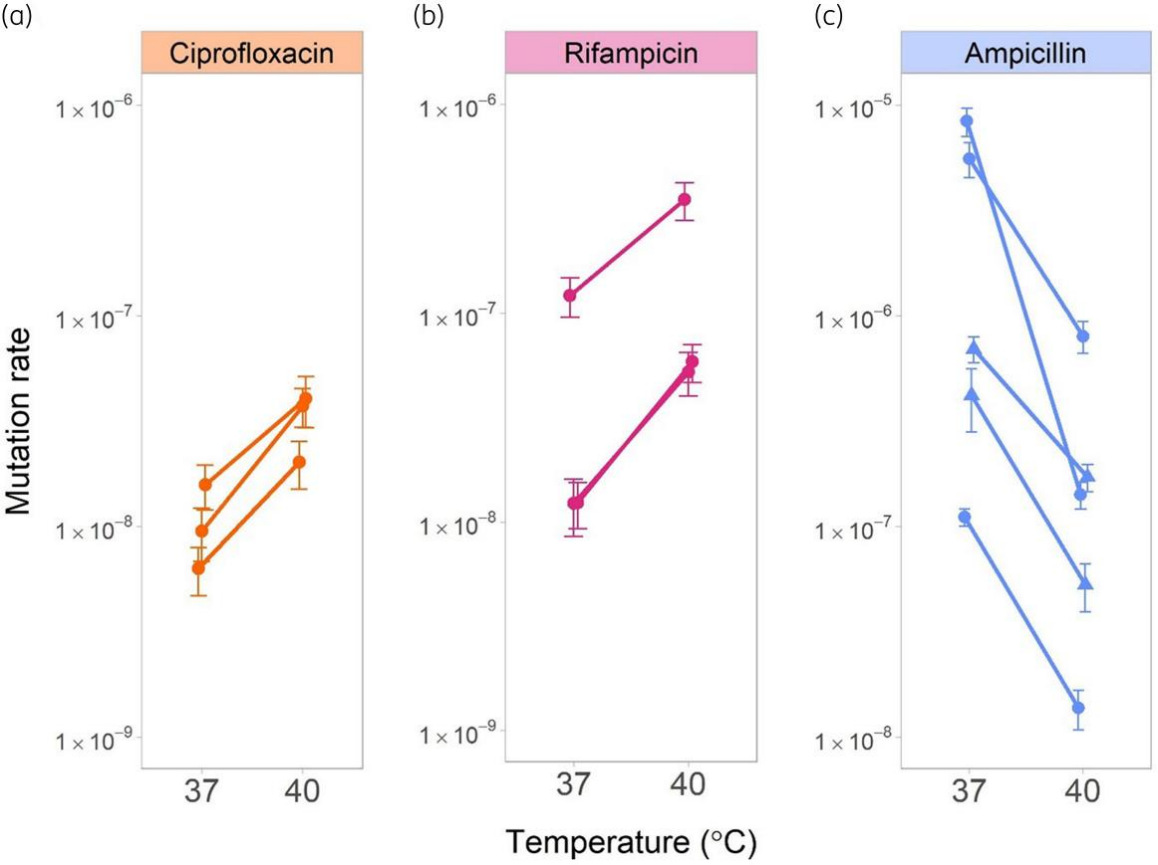
Effect of temperature on the mutation rate towards antibiotic resistance. At least three replicate experiments were conducted for each of the three antibiotics: (a) ciprofloxacin, (b) rifampicin and (c) ampicillin. Points show the estimated mutation rate for a given experiment and temperature, with error bars corresponding to  ± 1 standard error. Lines join the result for 37°C and 40°C for a single experiment; the difference between the mutation rate at 37°C and 40°C was statistically significant for all experiments (for ciprofloxacin the three *P* values ranged between 0.001 and 0.035; for rifampicin, the three *P* values ranged between 0.0002 and 0.003; for ampicillin, the five *P* values were all below 0.009). Circular points are for experiments performed at the University of Groningen, Netherlands; triangular points are for those performed at the University of Montpellier, France (protocol and sample size for these experiments deviate slightly, see Supplementary material 1.3). The estimation of population sizes was based on cfu counts. We used the estimation method maximum likelihood, and the Luria–Delbrück exponential lifetime model (default settings in FLAN).^17^

As shown in Figure 1, the mutation rate depended on temperature, yet the direction of this dependency differed between antibiotics. The mutation rate towards ciprofloxacin and rifampicin resistance was higher at 40°C than at 37°C. In contrast, the mutation rate towards ampicillin resistance was higher at 37°C than at 40°C. The patterns of temperature dependence were for each antibiotic robust to using different methods for quantifying mutation rates, including different estimators of final population size (see Supplementary material 2.4) and different estimation methods (see Supplementary material 2.5).

### Sequencing

A subset of mutants (3 per temperature per antibiotic, 18 in total) were fully sequenced. We identified mutations in known resistance genes for most mutants (13/18; see Supplementary material 2.3).

### Temperature-dependent antibiotic efficacy

To determine whether the observed changes in mutation rate (i.e. the rate of appearance of resistant phenotypes) were due to temperature-dependent antibiotic efficacy, we assessed antibiotic efficacy at the two experimental temperatures (see Supplementary material 2.6). For ampicillin, we found that it was consistently more effective at 40°C than 37°C. For rifampicin, the results were inconclusive. For ciprofloxacin, there was no consistent effect of temperature on antibiotic efficacy. Instead, the efficacy increased slightly when the protocol included a temperature change. In our fluctuation-test protocol, such a change in temperature does not occur, therefore this does not impact our mutation rate estimates.

## Discussion

We found that the mutation rate towards antibiotic resistance was temperature-dependent. A 3°C temperature change, such as the one associated with fever, altered the rate at which resistant mutants appeared by almost an order of magnitude for all three antibiotics considered. All else being equal, a higher rate of appearance of mutants with a resistant phenotype (mutation rate towards antibiotic resistance) makes evolutionary rescue more probable.^2^ This would thus increase the probability that a bacterial population successfully evolves resistance, which could lead to treatment failure.^1^ Furthermore, the timeframe in which resistance evolves will be shorter. These effects should be especially pronounced if the standing genetic variation at the start of infection is low, as would be expected due to the usually small size of the initial inoculum.^2,18^ Besides the mutation rate, other parameters that determine the probability of evolutionary rescue, such as the rate of population decay, may also be affected by temperature, an avenue for further study.^2^

For ciprofloxacin and rifampicin, we found that fever temperatures led to an increase in the mutation rate. These findings are consistent with the general finding that mutation rates are higher under suboptimal conditions. However, there may be an alternative explanation for rifampicin, as some mutations that confer rifampicin resistance also confer adaptation to elevated temperatures (see Supplementary material 2.3). Stress-induced mutagenesis could be an evolved response to facilitate evolutionary rescue under stressful conditions, but could also be a side effect of the fact that all biochemical processes (including those leading to mutation) are accelerated at higher temperatures.^6,7^ Regardless of the mechanism, all else being equal, resistance towards these antibiotics would be more likely to evolve under fever temperatures.

For ampicillin, contrary to our expectations, fever temperatures led to a decrease in the mutation rate towards resistance. This result was replicated in two different labs (Groningen and Montpellier), demonstrating its robustness. The observed effect of temperature on the mutation rate (i.e. rate of appearance of resistant phenotypes) could be due to a difference in the rate at which mutations appear in the genome (genomic mutation rate) or to a difference in the proportion of genomic mutations that convey resistance. The proportion of mutations that convey resistance could be influenced by temperature if the efficacy of an antibiotic is temperature-dependent (i.e. mutations that convey resistance at one temperature do not convey resistance at another temperature). Therefore, we conducted an experiment which showed an increase in the efficacy of ampicillin at 40°C (see Supplementary material 2.6, including results for ciprofloxacin and rifampicin). The increased efficacy of ampicillin at higher temperatures provides a likely explanation for the surprising results for ampicillin: some of the mutations conveying resistance at 37°C do not do so at 40°C, leading to a decrease in the rate of occurrence of resistant mutants. A study by Cruz-Loya *et al*.^19^ also found indications for the temperature-dependent efficacy of ampicillin. They hypothesized that this may be due to a synergistic effect between antibiotic-induced cell wall damage and the increased membrane permeability that occurs at higher temperatures. Regardless of the exact mechanism of temperature dependence, our experiments demonstrate that the rate of appearance of mutants resistant to ampicillin is temperature dependent, possibly due to the temperature-dependent efficacy of the antibiotic. Therefore, all else being equal, ampicillin resistance is less likely to evolve under fever temperatures.

The difference in the mutation rates observed at 37°C and 40°C is consistent between different repeats of the same experiment, and for ampicillin even showed replicability across laboratories. However, for all antibiotics, the estimates of the mutation rate differed considerably between replicate experiments. We speculate that these differences resulted from small variations (across replicates) in environmental conditions in the laboratory or small differences in the physiological state of the preculture cells from which the dilute cell suspension was made.

It would be worthwhile in the future to investigate how the current *in vitro* results translate to the *in vivo* context, as fever has wider effects than merely increasing temperature (such as stimulating immune function).^20^ There have been randomized controlled trials evaluating the impact of fever suppression in ICU patients receiving antimicrobial therapy, most notably by Young *et al.*^21^ Our results suggest that in the future such studies should account for the particular type of antibiotic administered. Future studies should also evaluate how our results translate to different antibiotics and bacterial species. Finally, the antibiotic concentrations considered were chosen to maximize the detection of changes in the mutation rate and are therefore lower than those used in a clinical setting; future work should reveal whether this pattern holds with higher, clinically relevant concentrations.

The temperature dependence of mutation rates towards antibiotic resistance implies that the evolution of antibiotic resistance (i.e. evolvability in the context of antibiotic resistance) is influenced by temperature. This study opens a new avenue to combat the emergence of antibiotic resistance: decisions regarding fever suppression could be leveraged to minimize the mutation rate to slow down or prevent the evolution of antibiotic resistance.

## Supplementary Material

dlae085_Supplementary_Data

## Data Availability

The data supporting the findings of this study are available at: https://doi.org/10.34894/BG4ZS8.
