# Supplementary material for: Temperature dependence of the mutation rate towards antibiotic resistance
Source: JAC Antimicrob Resist. 2024 Jun 6;6(3):dlae085. doi: 10.1093/jacamr/dlae085 (PMC11154133; doi:10.1093/jacamr/dlae085)
Supplement: dlae085_Supplementary_Data [file dlae085_supplementary_data.docx]

**Temperature dependence of the mutation rate towards antibiotic resistance**

Timo J.B. van Eldijk, Eleanor A. Sheridan, Guillaume Martin, Franz J. Weissing, Oscar P. Kuipers, G. Sander van Doorn

Supplementary material

# Contents

| 1. Detailed methods |  | Page |
| --- | --- | --- |
|  | - 1. Strain & media | 2 |
|  | - 1. Fluctuation test | 2 |
|  | - 1. Fluctuation test ampicillin conducted in Montpellier | 3 |
| 1. Supplemental results |  |  |
|  | - 1. Comparing growth rates | 4 |
|  | - 1. Comparing plating efficiency | 6 |
|  | - 1. Sequencing results | 7 |
|  | - 1. Estimation of mutation rates using CFP fluorescence | 9 |
|  | - 1. Different procedures for estimating mutation rates | 11 |
|  | - 1. Temperature-dependent antibiotic efficacy | 14 |
| 1. References |  | 18 |

# Detailed Methods

### 1.1 Strain & media

The bacterial strain used in this experiment was *Escherichia coli* REL4548-CFP-lux. The ancestor of this strain was isolated in the long-term evolution experiment where *E. coli* REL606 adapted for 10000 generations to DM25 medium (Lenski et al., 1991; Lenski & Travisano, 1994; Elena et al. 1998). Subsequently, this strain was transformed with a chromosomally integrated constitutive high expression CFP marker (Gallet et al. 2012) and kindly provided by Romain Gallet. Subsequently, this strain was transformed with a chromosomally integrated lux reporter genes according to the protocol described by Howe et al. (2010). The plasmid used for the chromosomal integration of lux reporter genes can be found here <https://www.addgene.org/69150/>. The resulting strain REL4548-CFP-lux, which we used in our experiment, constitutively expresses CFP and lux reporter genes, allowing for close monitoring of cell density and metabolic activity respectively.

CFP fluorescence was measured using a BMG Labtech CLARIOstar with excitation = 431 ± 15 nm and emission = 472 ± 15nm. All experiments were conducted using Davis minimal media containing 1 mg/mL glucose (DM1000). For plating, this medium was supplemented with 1.6% (w/v) agar and, when required, antibiotics were added to give the following final concentrations: rifampicin 10 μg/mL, ciprofloxacin 0.013 μg/mL, and ampicillin 1.5 μg/mL. These concentrations were chosen by plating 200 μL of culture on a range of antibiotic concentrations and selecting a concentration that resulted in a countable number of mutants, thereby maximizing the power to detect changes in the mutation rate. Rifampicin stocks were made by dissolving rifampicin (Sigma) in 0.1N HCl; ciprofloxacin stocks were made by dissolving ciprofloxacin (Sigma) in 0.1N HCl; and ampicillin stocks were made by dissolving ampicillin sodium (Sigma) in sterilized distilled water.

### 1.2 Fluctuation test

To measure the mutation rate at two different temperatures, a fluctuation test was performed. To set up the experiment, *E. coli* REL4548-CFP-lux was grown to stationary phase overnight at 37 °C (incubator: INCU-line, IL 56 Premium; agitation: Grant-Bio PMS 1000i, shaking = 540 PM). For the ampicillin fluctuation tests, the stationary-phase culture was diluted with a factor 10^6^ to create the diluted cell suspension needed to initialize the fluctuation test (approximately 100 cells per population). For ciprofloxacin and rifampicin, the stationary-phase culture was refreshed after approximately 24 hours. This was done by diluting the culture with a factor 10 and growing it for another 2.5 hours at 37 °C. Refreshing the overnight culture ensured that all cells were in the exponential growth phase prior to the start of the experiment, minimizing the lag time and thereby reducing possible differences in final population densities caused by stochastic variations in lag time. The refreshed culture was then adjusted to an OD_600_ value of 0.5 and diluted with a factor 2.5 x 10^5^, creating the diluted cell suspension needed to initialize the fluctuation test (approximately 200 cells per population).

In all experiments, the diluted cell suspension was then used to initialize the fluctuation test, by establishing 40 populations in each of three 96-well plates (Greiner bio one, clear), for a total of 120 populations. To minimize the effect of evaporation only the inner wells of the plate were used, with the wells bordering the edge of the plate filled with medium. Each population consisted of 200 μL of diluted culture, with each population initially consisting of approximately 100 cells for the ampicillin experiments and approximately 200 cells for rifampicin and ciprofloxacin experiments. These initial cell numbers per population are somewhat higher than the single cell assumed by the fluctuation test, yet they are low enough to ensure that the probability that mutations are present at the start of the experiment is small (hence ensuring statistical independence between resistance emergence events). These somewhat higher initial cell numbers allow to limit stochastic population extinction upon starting lineages, and to limit the differences in the final population density (after a fixed growth period) caused by early stochastic growth dynamics. The first 96-well plate was used to monitor cell growth in a plate reader (shaking = 400 RPM, double orbital). This monitor plate served as a rough indication of when the populations were in mid-exponential phase, this plate was not directly used to gather any data. The other two plates were grown under agitation (Grant-Bio PMS 1000i; ciprofloxacin and rifampicin shaking = 700 RPM; ampicillin shaking = 540 RPM) in identical incubators (INCU-line, IL 56 Premium) at 37 °C and 40 °C respectively.

When the populations were roughly in mid-exponential phase (determined using CFP fluorescence) the number of mutants in each population was assessed by plating the entire population on agar plates containing antibiotics. These agar plates were incubated for five or six days at 37 °C or at 40 °C (at the temperature at which the populations were grown in the 96-well plate). Subsequently, the number of mutants on each agar plate was counted. The final population densities at the time of plating were determined in two ways: through CFU-counting and CFP fluorescence measurements. For CFU counting a 2 μL sample was taken from a subset of 12 of the populations (for one of the AMP experiments a subset of 4 populations was used), this sample was subsequently diluted with a factor of 10^5^ and plated on agar plates without antibiotics, this provided an estimate of the mean final population densities. For CFP fluorescence all replicate populations were measured in plate reader (BMG Labtech CLARIOstar, excitation = 431 ± 15 nm, emission = 472 ± 15nm), and using a calibration curve the final number of cells for each replicate population could be estimated. This experiment was repeated three times for each antibiotic in Groningen (NL).

The R-package FLAN (Mazoyer et al. 2016) was used to analyze the data, using the “mutestim” function to compute mutation rate estimates and using the “flan.test” function to perform a fluctuation analysis test to compare the mutation rate between the two temperatures. Several different estimation methods and lifetime models implemented in the FLAN package were used.

### 1.3 Fluctuation tests ampicillin conducted in Montpellier

For ampicillin, two additional fluctuation test experiments were conducted in Montpellier (FR). The experiments shown are a subset of pilot experiments used to create a fine-tuned protocol, hence the protocol for these two experiments deviates slightly from the one described above for the experiments conducted in Groningen. Nonetheless, these experiments are included as they illustrate that the patterns observed for ampicillin replicate across two different labs.

First of all, populations created from the dilute cell suspension had a culture volume of 250 μl. To determine the population densities only a subset of 8 populations (instead of 12) were sampled, diluted with a factor 10^5^ and plated on agar plates without antibiotics. No CFP measurements of final population density were performed. For the first ampicillin experiment conducted in Montpellier the experiments for the two temperatures 37 °C and 40 °C were conducted on two different days, the number of replicate populations also differed between these two days. For 37 °C, 14 populations were plated on antibiotic plates. For 40 °C, 30 populations were plated on antibiotic plates. For the second ampicillin experiment conducted in Montpellier the experiments for the two temperatures, 37 °C and 40 °C , were performed on the same day. For each temperature a total of 52 populations was plated on antibiotic plates to asses the number of mutants in each population.

# Supplemental results

Several additional experiments were conducted to verify that some of assumptions of the fluctuation test experiments hold. This included comparing the growth rates at 37 °C and 40 °C and comparing the plating efficiency at 37 °C and 40 °C. Additionally, a limited set of mutants was fully sequenced to confirm that the colonies growing on antibiotic containing plates do indeed represent genetic mutants. Furthermore, the mutation rates were estimated using CFP fluorescence as a measure of final population density (instead of CFU counting). In addition, the mutation rates were estimated using several different estimation methods and lifetime models implemented in the FLAN package. Finally, an experiment was conducted to assess temperature-dependent antibiotic efficacy.

### 2.1 Comparing growth rates

A fluctuation test assumes a pure birth process, hence differences in growth rates between the two treatments can cause discrepancies (i.e., a different total number of divisions between the treatments). Therefore, the growth rate of *E. coli* REL4548-CFP-lux was at measured at 37 °C and 40 °C using CFP fluorescence. An overnight culture was diluted 10^6^-fold and was used to create populations in a 96-well plate in a similar way to the fluctuation test. This plate was subsequently grown until stationary phase in the plate reader. This was repeated on two separate days: on the first day the 96-well plate was incubated at 37 °C, and on the second day the 96-well plate was incubated at 40 °C . To prevent any effect of evaporation on the wells on the edge of 96-well plate were excluded from the analysis, resulting in 54 replicate populations for each temperature. To compare growth rates between the two temperatures the linear portion of each population’s log density dynamics (corresponding to exponential growth) was plotted (figure S1). Visual inspection as well as a statistical test (figure S2) indicated that the growth rate was not different between 37 °C and 40 °C (*p*= 0.7881).


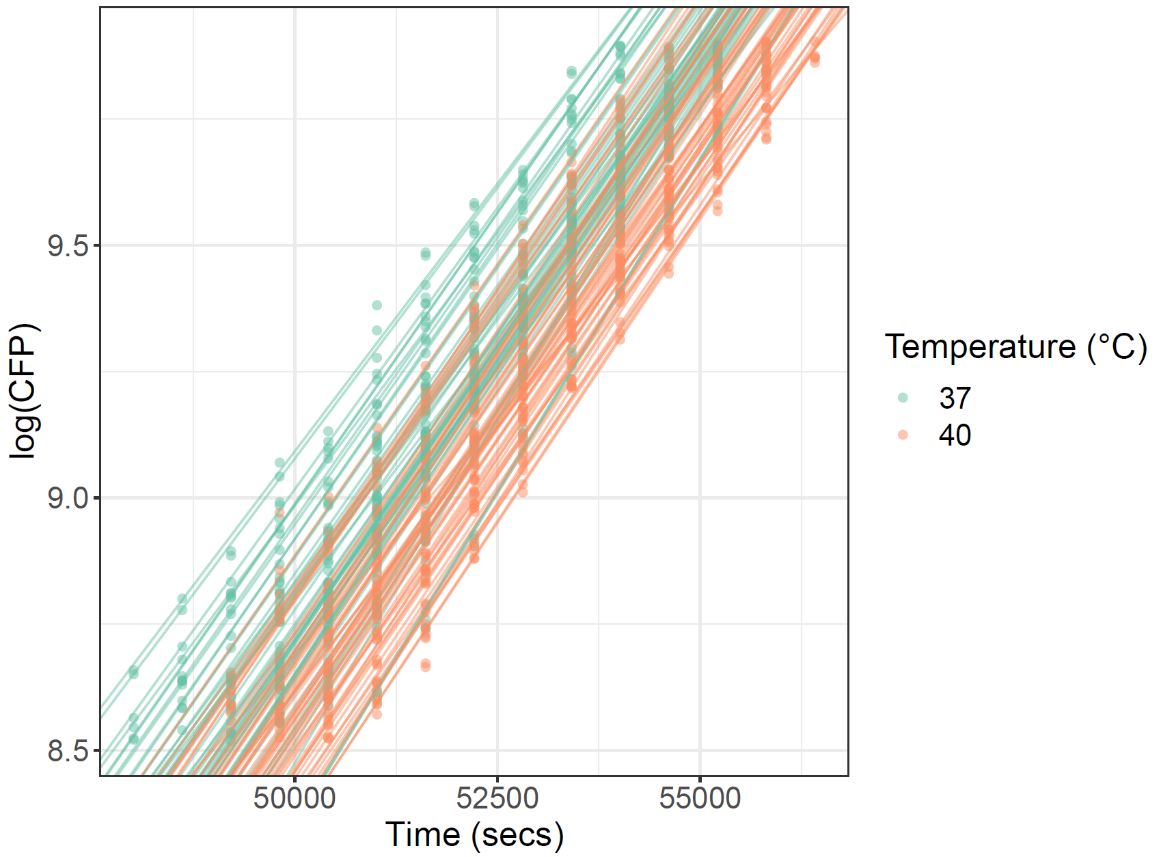


**Figure S1.** Linear portion of the log density dynamics (log (CFP)), including the linear models fit to these dynamics.


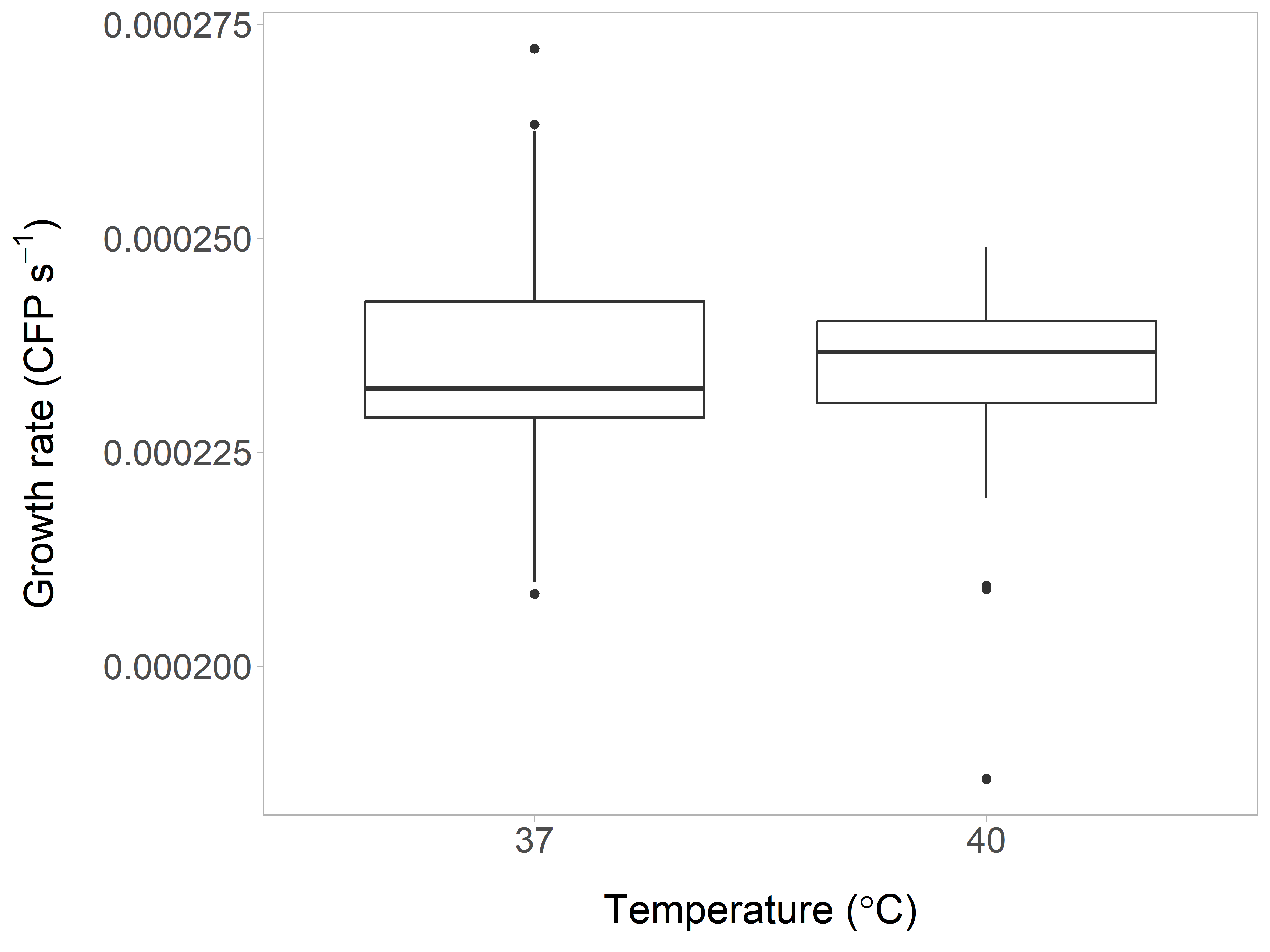


**Figure S2.**  Temperature did not significantly affect growth rate. The linear portion of the log density dynamics was extracted and the slopes compared between temperatures using a *t*-test (*p*= 0.7881). Vertical line shows the median, box shows the interquartile range.

### 2.2 Comparing plating efficiency

Differences in plating efficiency between the two different temperatures could cause discrepancies in estimating final population densities. Therefore, the plating efficiency of cells plated on standard agar plates (without antibiotics) was compared between the two temperatures. Specifically, an overnight culture was grown to stationary phase prior to being diluted 10^6^-fold. 25 μl of this dilution was then plated on 40 non-selective DM1000 agar plates. Subsequently, half of the plates were grown at 37 °C and half at 40 °C, resulting in 20 replicates per temperature. Plates were photographed and counted as for the fluctuation test. The resulting data is shown in figure S3, a Wilcoxon rank sum test showed no significant difference in plating efficiency between 37 °C and 40 °C (*p* = 0.3935).


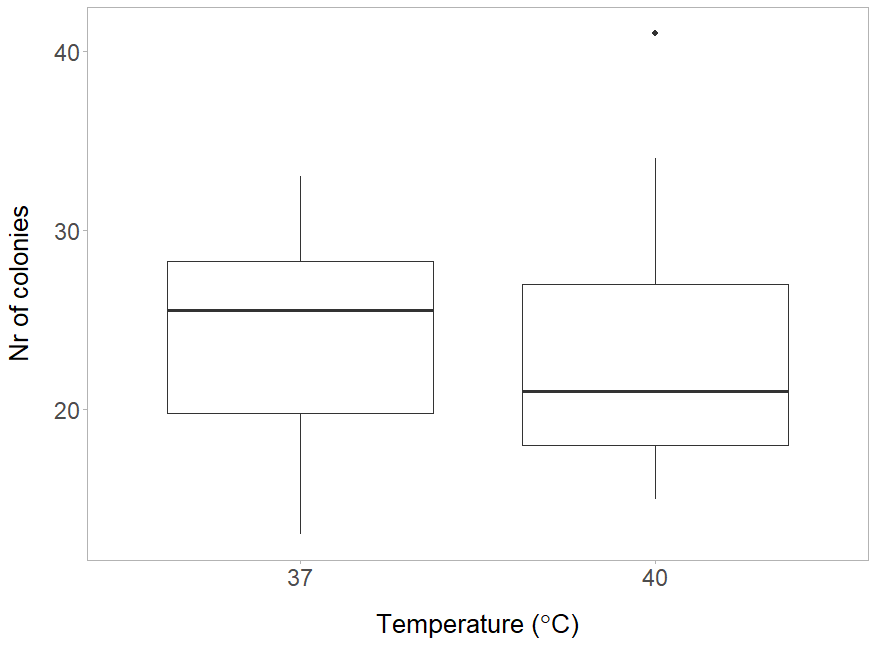


**Figure S3.**  Comparing the plating efficiency of cells plated on standard agar plates (without antibiotics) between the two temperatures 37 °C and 40 °C. Vertical line shows the median, box shows the interquartile range.

### 2.3 Sequencing results

To see if the colonies observed on the agar plates with antibiotics do indeed have mutations conferring resistance, a small subset of mutants was fully sequenced (three per temperature per antibiotic, 18 in total) and their genomes were screened for relevant resistance mutations. Subsequently, the mutants and the wild-type REL4548-CFP-lux strain were grown in DM1000 and DNA was extracted using the GenElute Bacterial Genomic DNA Kit (Merck). The extracted DNA was quantified using a nanodrop and subsequently shipped to BGI Europe for sequencing, where sequencing was performed on a DNBseq machine using a read length of 150 bp, sequencing about 10 million reads per sample. The resulting reads for the starting strain and the mutants were aligned to the REL606 reference sequence (NC_012967.1, resulting in approximately 300X coverage) and the mutations were analysed using Breseq (Detherage & Barrick, 2014). The differences between the reference and the starting strain were subtracted from those in the mutants so that only mutations that occurred during the experiment were observed. The results are summarized in Table S1. Mutations in known resistance-associated genes were identified in 13/18 mutants tested.

We did not find any clear association between the growth temperature of the mutants and particular types of mutations for ampicillin and ciprofloxacin. For rifampicin, we see that two out of three rifampicin-resistant mutants grown at 40 °C possess a mutation in the I572N codon of the *rpoB* gene. Whilst none of the mutants grown at 37 °C possess a mutation in this particular codon (instead they have other mutations in the *rpoB* gene). However, the number of mutants sequenced is too low to confidently infer a pattern. Interestingly, based on data by Rodríguez-Verdugo et al. (2013) a mutation in the I572N codon provides both rifampicin resistance as well as adaptation to an elevated temperature. Selection for this particular mutant at higher temperatures could provide an alternative explanation for the increased rate of appearance of rifampicin-resistant mutants at fever temperatures. Note that even if such selection drives the observed increase in the rate of appearance of resistant mutants at elevated temperatures, fever suppression should still be an effective strategy to mitigate the evolution of rifampicin resistance as it would decrease selection for these mutations.

**Table S1.** Resistance mutations were identified using Breseq in fully sequenced mutants and references to sources identifying and studying similar mutations.

| Anti-  biotic | Temper-  ature | Nr | Mutation | Position | Gene mutated | Citation |
| --- | --- | --- | --- | --- | --- | --- |
| Amp | 37 | 1 | A🡪C  L350F (TTA→TTC) | 95,266 | Peptidoglycan glycosyltransferase FtsI (*ftsl*) | Li et al. 2019 |
| Amp | 37 | 2 | G🡪A  L714L (CTG→CTA) | 433,084 | Endopeptidase La (*lon*) | Nicoloff et al. 2013 |
| Amp | 37 | 3 | G🡪T  E96D (GAG→GAT) | 3,464,981 | 2‑component system response regulator OmpR (*ompR*) | Jordan et al. 2022 |
| Amp | 40 | 1 | +AA coding (498/1089 nt) | 1,003,577 | porin OmpF (*ompF*) | Jordan et al. 2022 |
| Amp | 40 | 2 | - | - | - | - |
| Amp | 40 | 3 | Δ2 bp coding (264‑265/1353 nt) | 3,464,288 | 2‑component system sensor histidine kinase EnvZ (*envZ*) | Jordan et al. 2022 |
| Cipro | 37 | 1 | - | - | - | - |
| Cipro | 37 | 2 | - | - | - | - |
| Cipro | 37 | 3 | Δ1 bp coding (773/1089 nt) | 1,003,302 | porin OmpF (*ompF*) | Vinue et al. 2015;  Kishi & Takei 2009; Forst et al., 1989 |
| Cipro | 40 | 1 | - | - | - | - |
| Cipro | 40 | 2 | Δ9 bp intergenic (‑9/+585) | 1,004,083 | porin OmpF/asparagine‑‑tRNA ligase (*ompF/asnS*) | Vinue et al. 2015; Kishi & Takei 2009; Forst et al., 1989 |
| Cipro | 40 | 3 | Δ314 bp | 3,464,348 | 2‑component system response regulators (*envZ, ompR*) | Vinue et al. 2015; Kishi & Takei 2009; Forst et al., 1989 |
| Rif | 37 | 1 | T→C  I530T  (ATC→ACC) | 4,162,444 | DNA‑directed RNA polymerase subunit beta (*rpoB*) | Weinstein & Zaman 2018 |
| Rif | 37 | 2 | G→T  R529L (CGT→CTT) | 4,162,441 | DNA‑directed RNA polymerase subunit beta (*rpoB*) | Weinstein & Zaman 2018 |
| Rif | 37 | 3 | - | - | - | - |
| Rif | 40 | 1 | T→A  I572N (ATC→AAC) | 4,162,570 | DNA‑directed RNA polymerase subunit beta (*rpoB*) | Weinstein & Zaman 2018 |
| Rif | 40 | 2 | T→A  I572N (ATC→AAC) | 4,162,570 | DNA‑directed RNA polymerase subunit beta (*rpoB*) | Weinstein & Zaman 2018 |
| Rif | 40 | 3 | T→A  L511Q (CTG→CAG) | 4,162,387 | DNA‑directed RNA polymerase subunit beta (*rpoB*) | Weinstein & Zaman 2018 |

### 2.4 Estimation of mutation rates using CFP fluorescence

A calibration curve was constructed to translate CFP fluorescence values to colony forming units (CFU). To construct this calibration curve, a stationary phase culture was repeatedly diluted two-fold and CFP emissions for each dilution read using the plate reader (BMG Labtech CLARIOstar, excitation = 431 ± 15 nm, emission = 472 ± 15nm, shaking = 400 RPM, double orbital). To collect CFU data, a separate dilution was made so that there was a sufficient volume of bacterial culture for plating. From this diluted culture, six replicates of each of three concentrations (0.5 x 10^-6^, 1 x 10^-6^, and 2 x 10^-6^) of 200 μL each were plated on non-selective DM1000 agar and grown at 37 °C for approximately 24 hours before counting as above. Since all of these dilutions gave a countable number of cells, each replicate was converted to the number of cells in an undiluted population and the mean calculated. This was then used to calculate the expected number of cells in each of the dilutions for which CFP emissions had been measured. A linear model was computed between CFU as estimated above and CFP emissions, giving a calibration curve (figure S4). Final population densities were also estimated using CFP fluorescence. The calibration curve is shown in figure S4 was then used to infer final population densities for the fluctuation test experiments, using CFP fluorescence measurements. The obtained results are shown in figure S5, as can be observed they are in agreement with those obtained when estimating final population density using classical CFU counting.


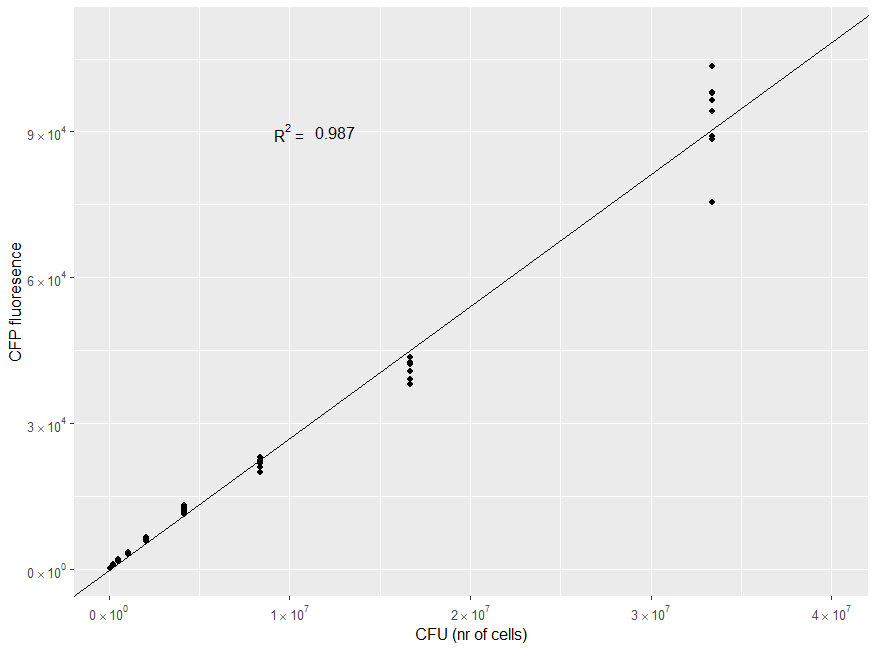


**Figure S4.** Calibration curve between CFP fluorescence and colony forming units (CFU)


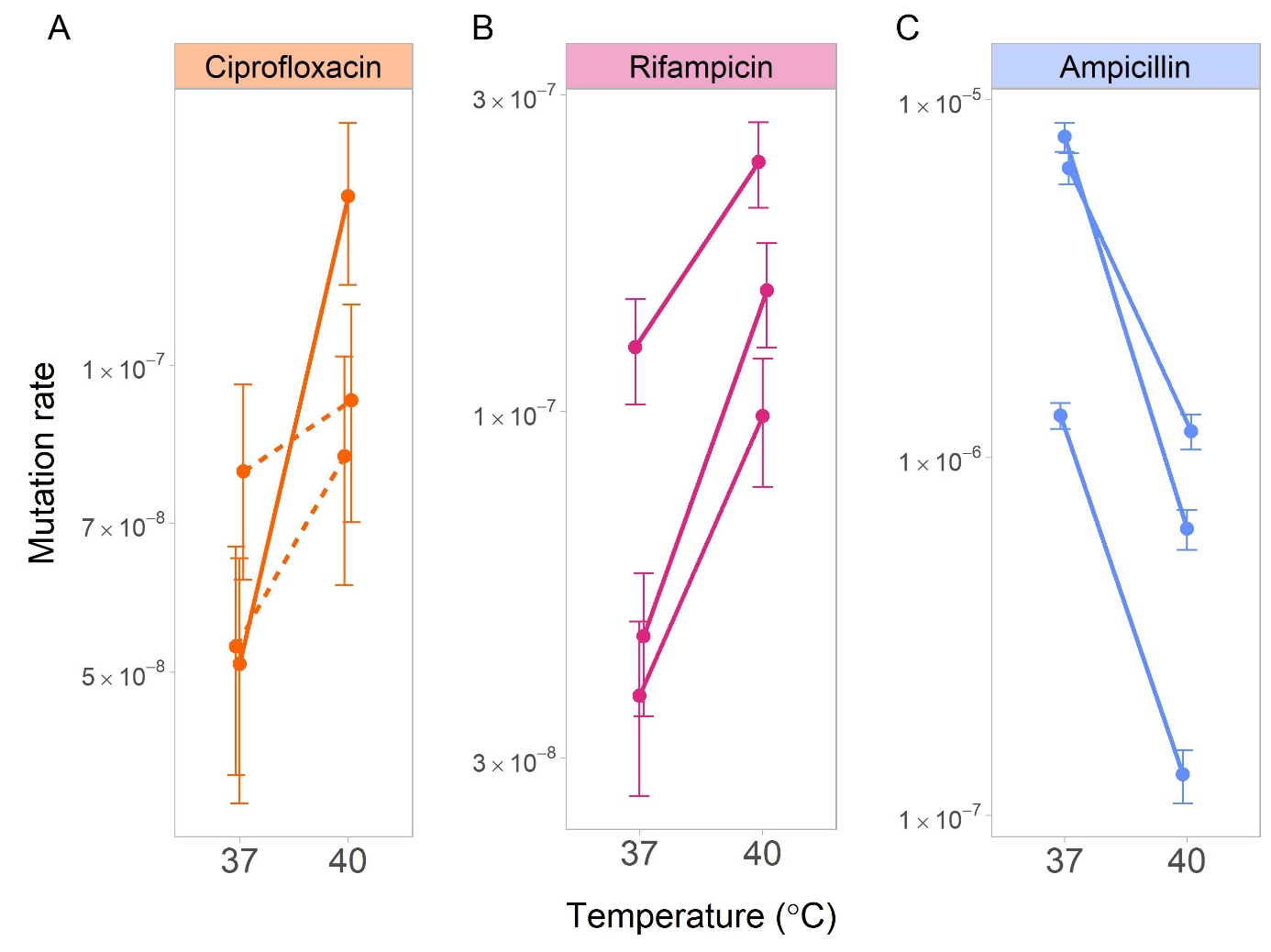


**Figure S5.**  Mutation rates estimated using CFP fluorescence to assess final population density for each population: **(A)** ciprofloxacin, **(B)** rifampicin, and **(C)** ampicillin. Points show the estimated mutation rate for a given experiment and temperature, with error bars corresponding to ± 1 standard error. Lines join the result for 37 °C and 40 °C for a single experiment; significant differences between mutation rate at 37 °C and 40 °C (*p* < 0.05) are shown as solid lines, non-significant differences are shown as dotted lines. All experiments shown were performed at the University of Groningen, Netherlands. The mutation rates were estimated using the FLAN package, using the estimation method maximum likelihood, and using the Luria-Delbrück exponential lifetime model (default settings).

### 2.5 Different procedures for estimating mutation rates

Several procedures for inferring mutation rates are implemented in the FLAN package (17). These include two different estimation methods (maximum likelihood and generating function) and three different lifetime models (Luria-Delbrück exponential lifetime model, Haldane constant lifetime model and Inhomogeneous model). Figure 1 in the main text is based on the default setting in FLAN (maximum likelihood, Luria-Delbrück model). Figures S6 to S10 show the results when applying the other combinations of estimation method and lifetime model to our data. The figures show that our conclusions are robust to the method chosen: in all cases, the differences between the mutation rate at 37 °C and 40 °C were in the same direction and significant for all experiments (*p* < 0.05).


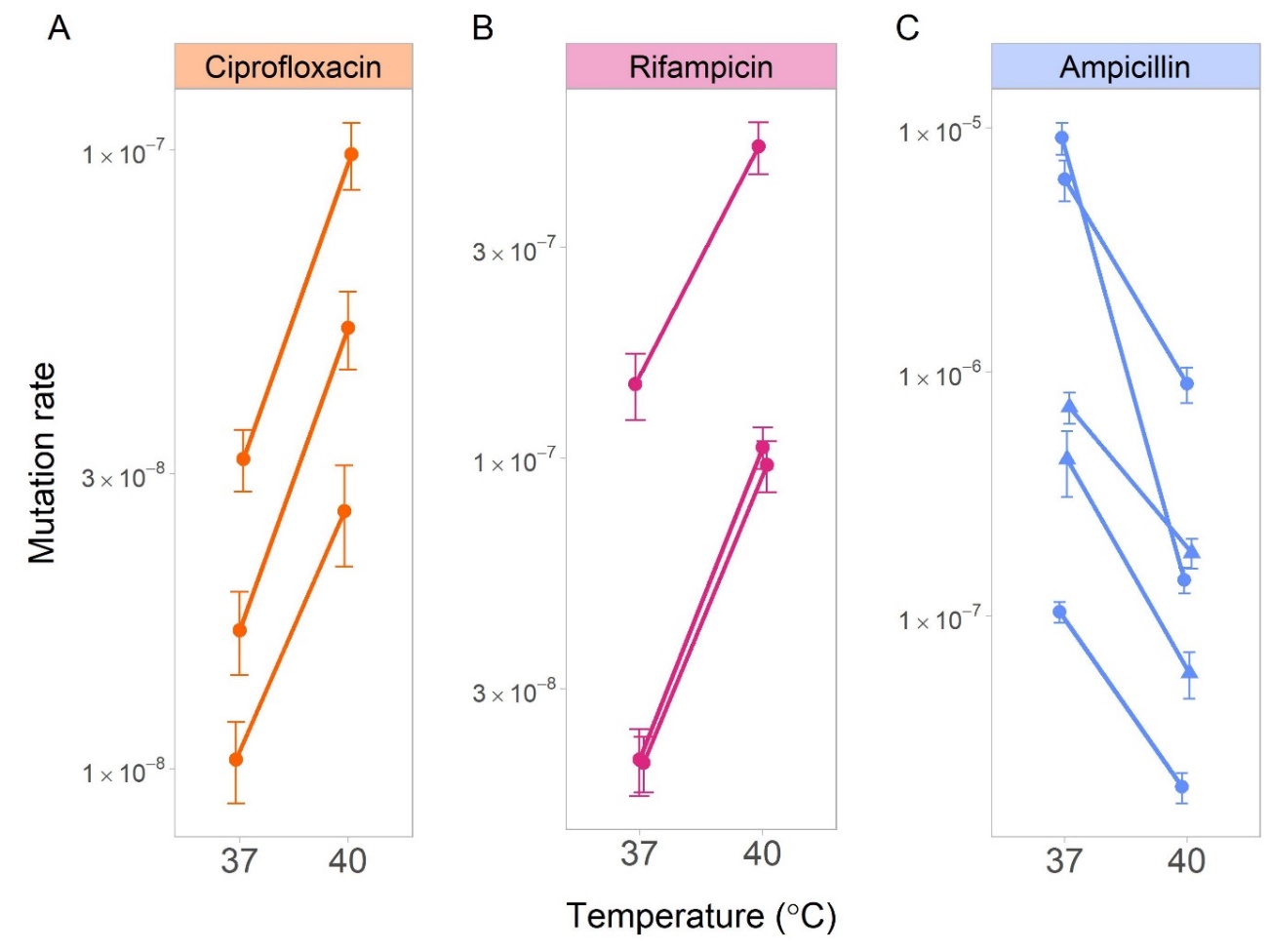


**Figure S6.** As Figure 1 in the main text, but using the estimation method maximum likelihood, and using the Haldane constant lifetime model.


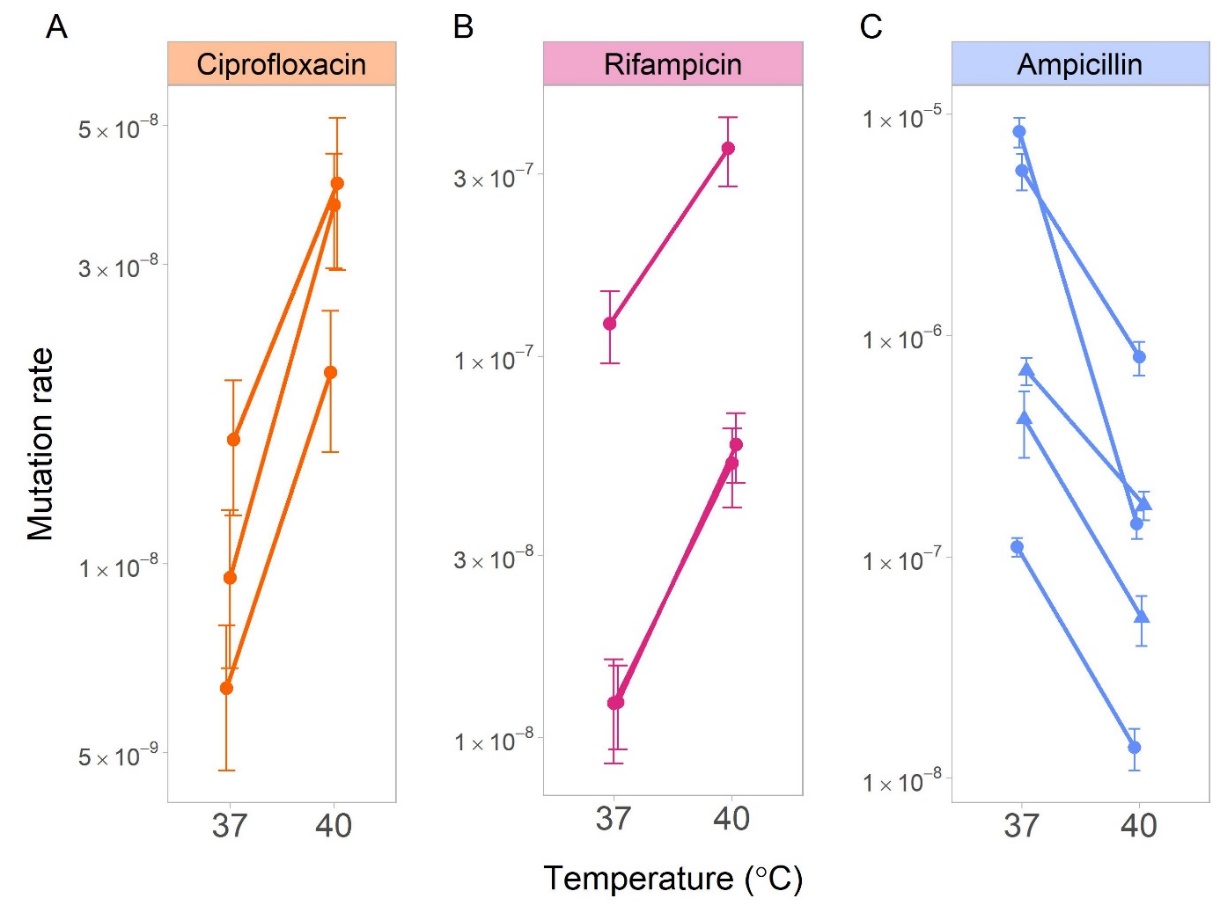


**Figure S7.**  As Figure 1 in the main text, but using the estimation method maximum likelihood, and using the inhomogeneous lifetime model.


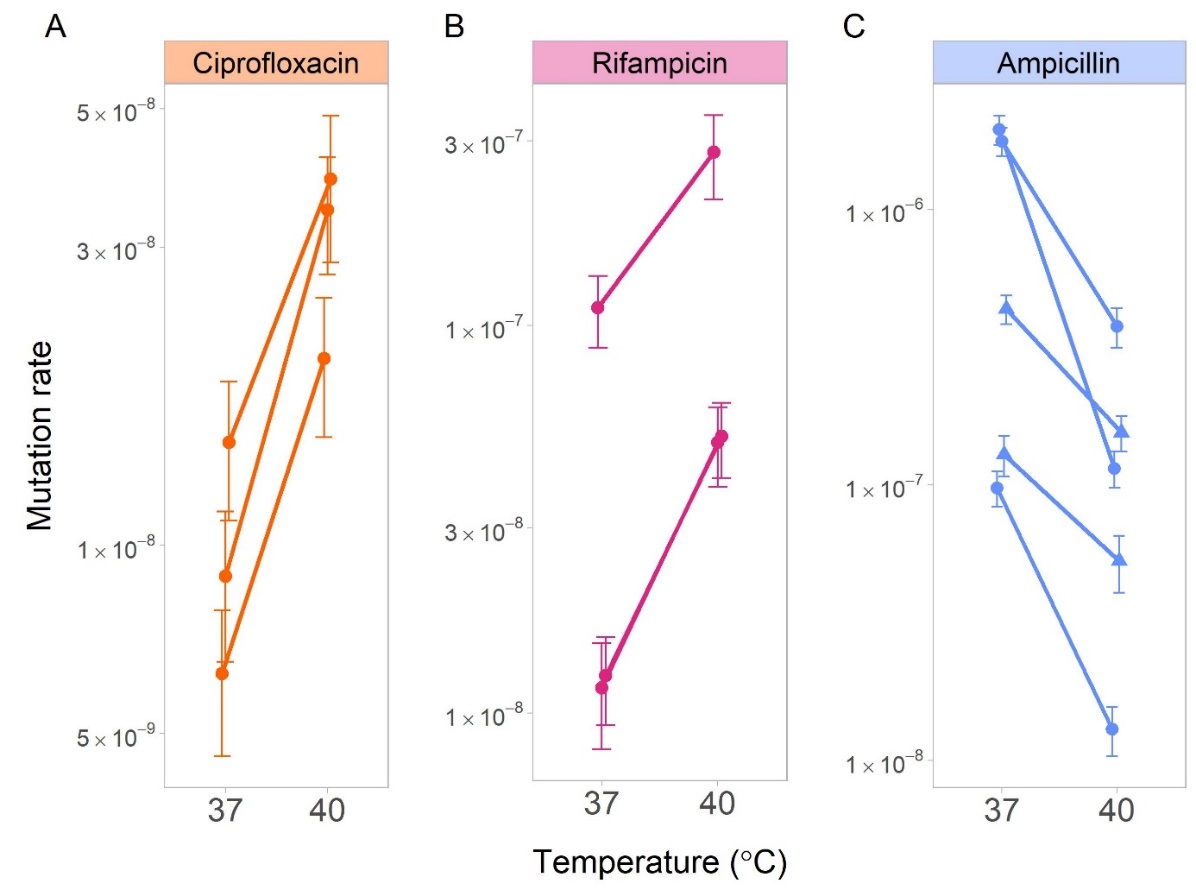


**Figure S8.** As Figure 1 in the main text, but using the estimation method generating function, and using the Luria-Delbrück exponential lifetime model.


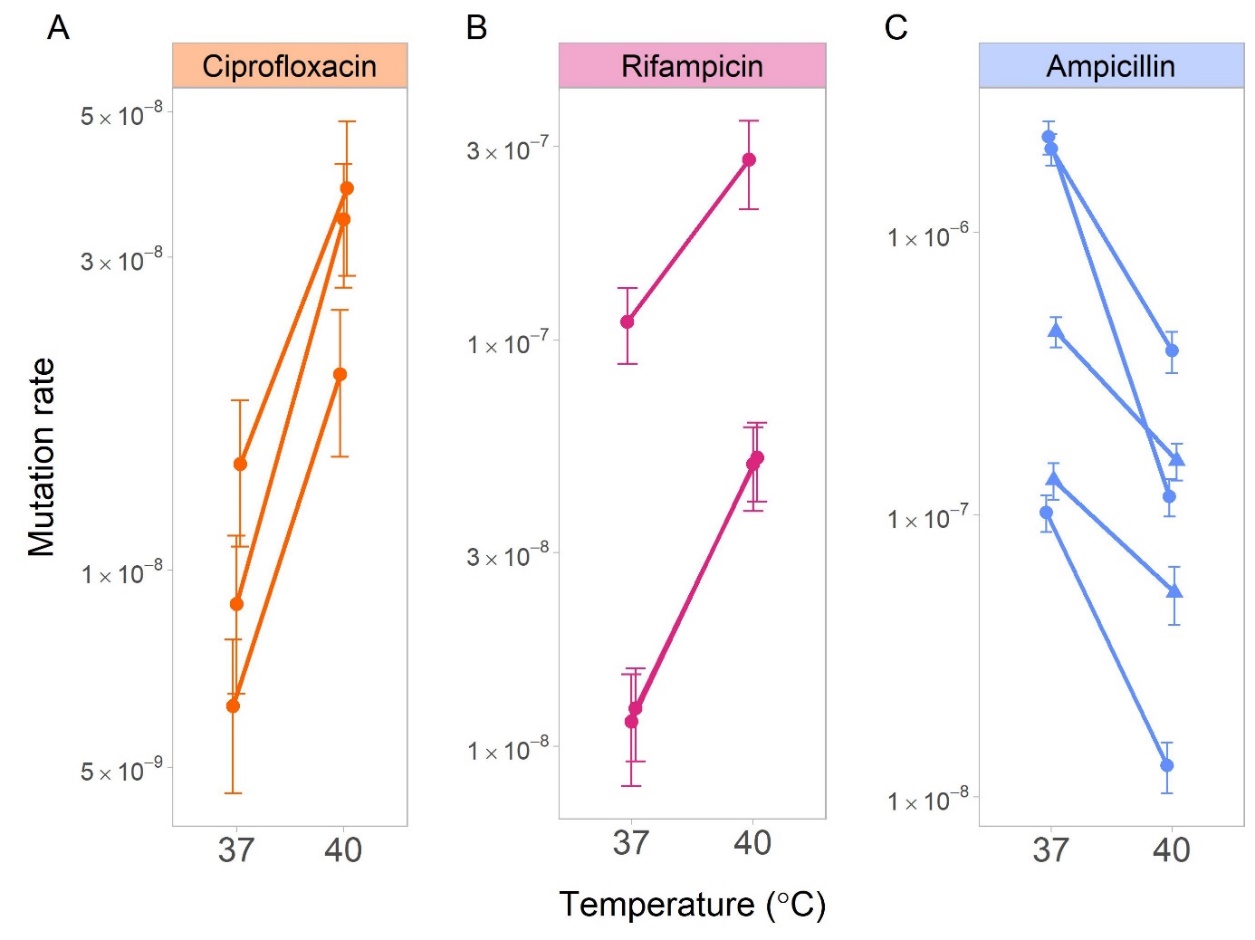


**Figure S9.**  As Figure 1 in the main text, but using the estimation method maximum likelihood, and using the Haldane constant lifetime model.


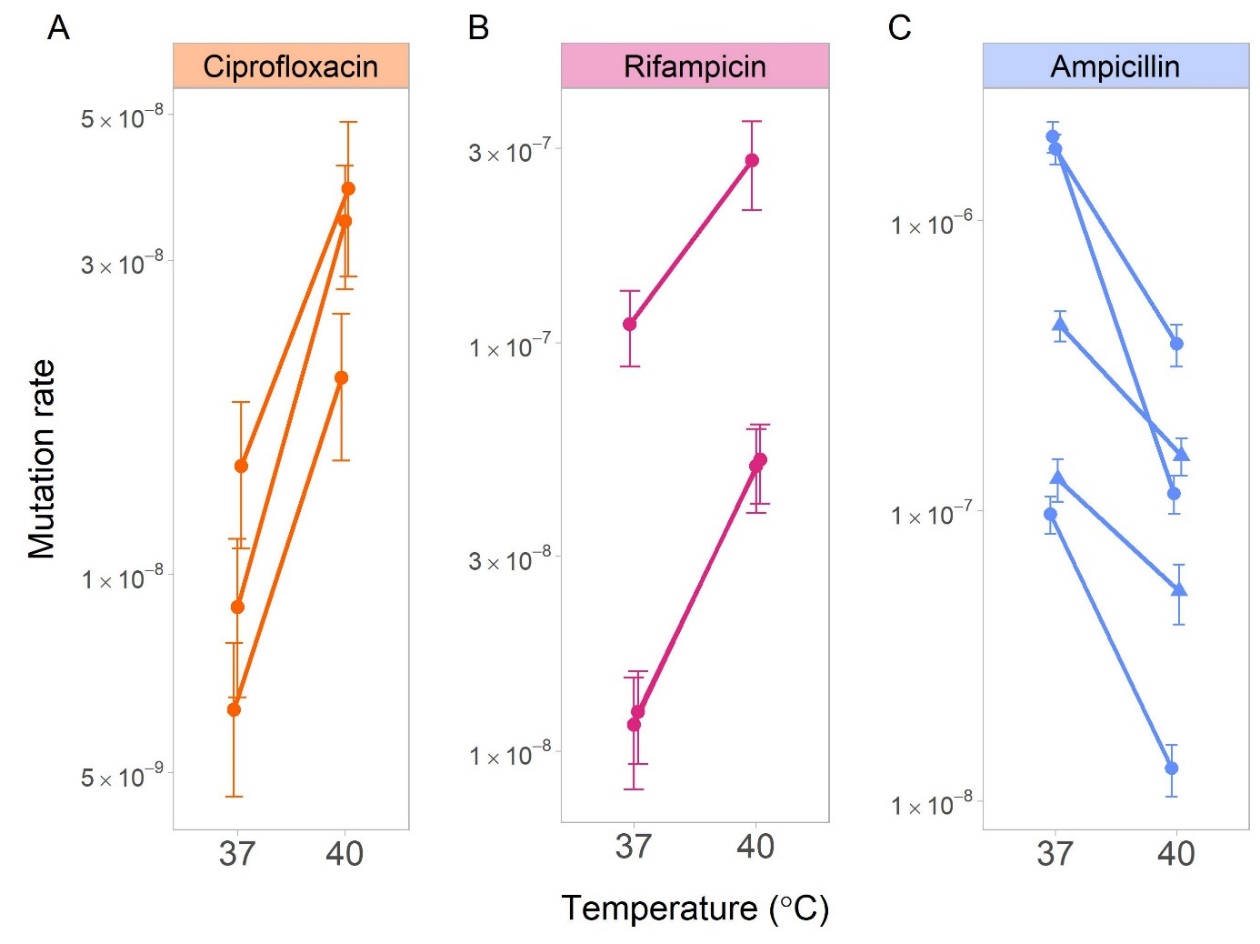


**Figure S10.**  As Figure 1 in the main text, but using the estimation method maximum likelihood, and using the inhomogeneous lifetime model.

### 2.6 Temperature-dependent antibiotic efficacy

Temperature-dependent changes in the mutation rate observed in the fluctuation test could be due to changes in the rate at which mutations occur in the genome (genomic mutation rate) or, alternatively, they could be caused by differences in the proportion of mutations that convey resistance at each temperature. The latter effect could be caused by the temperature-dependent efficacy of the antibiotic, implying that mutations that convey resistance at one temperature are not able to convey resistance at the other temperature. To explore the temperature-dependent efficacy of each of the three antibiotics, we conducted a fluctuation test as described above. However with an important change, that when assessing the number of resistant mutants in a population (grown in a 96-well plate in liquid culture at 37 °C or 40 °C), each population was split in half, and half of the population was grown on antibiotic-containing plates at 37 °C and the other half was grown on antibiotic-containing plates at 40 °C. Therefore, for each liquid culture temperature, there were two different antibiotic agar plate culture incubation temperatures. For each population, the number of mutants on each of the two antibiotic agar plates should be equal if there is no temperature-dependent efficacy of the antibiotic, and if we ignore the production of de novo mutations during growth on the agar plates, as is classically done in fluctuation test data analysis. If on the other hand the efficacy of the antibiotic is different per temperature, some of the mutants that are resistant at one temperature should not be resistant at another temperature, leading to a difference in the observed number of mutants on the two agar plates originating from the same liquid culture population. Only those liquid culture populations in which at least one mutant was detected were included in the analysis, since being able to detect the impact of antibiotic agar incubation temperature is conditional on the presence of mutants in the liquid culture populations. For each liquid culture temperature, the impact of antibiotic agar incubation temperature was assessed using a Wilcoxon signed-rank test.

For ciprofloxacin (figures S11 and S12) it was found that for both liquid culture temperatures, the antibiotic agar incubation temperature significantly impacted the number of mutants detected (for liquid culture 37 °C *n* = 22 and *p* = 0.0394, for liquid culture 40 °C *n* = 13 and *p* = 0.0422). However, as can be seen in figure S12, the sign of this effect was different for each liquid culture temperature. There is thus no consistent effect of temperature on the efficacy of ciprofloxacin, instead it seems that a change in temperature between the liquid culture phase and the growth on antibiotic agar increases the efficacy of ciprofloxacin. We hypothesize that a change in temperature causes a mild-stress on the cells, subsequently ciprofloxacin works more effectively to kill these mildly stressed cells. We note that such a change in temperature does not occur in our original fluctuation-test protocol as antibiotic agar plates are incubated at the same temperature as the liquid culture from which they originate. Therefore, the effect of a change in temperature on the efficacy of ciprofloxacin should not impact our mutation rate estimates obtained using the fluctuation test.

For rifampicin the sample size was unexpectedly small i.e. fewer than expected agar plates showed resistant mutants (for liquid culture 37 °C *n* = 2, for liquid culture 40 °C *n* = 4). The exact cause of this is unknown, however, it could be due to a small dosage error when adding the antibiotic to the agar plates: the experiments are quite sensitive to the exact concentration of the antibiotic in the agar plates. Due to the low sample size no statistical testing could be conducted, the data is shown in figure S13. For ampicillin (figures S14 and S15) a consistent effect of antibiotic agar incubation temperature was detected regardless of liquid culture temperature (for liquid culture 37 °C *n* = 17 and *p* = 0.0003, for liquid culture 40 °C *n* = 14 and *p* = 0.0024). This clearly indicates that the efficacy of ampicillin is temperature dependent, and/or that de novo mutations, on the plates, contribute to the outcome, and they are produced at different rates in different temperatures.

**
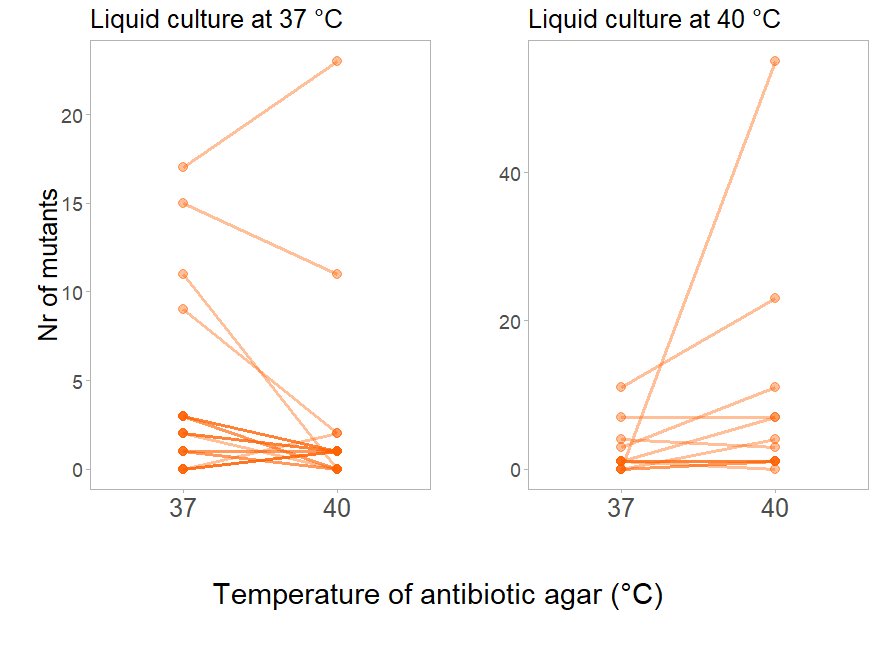
**

**Figure S11.**  The number of ciprofloxacin-resistant mutants detected in each population at each antibiotic agar incubation temperature, lines connect samples originating from the same liquid culture. The left panel shows populations grown in liquid culture at 37 °C. The right panel shows populations grown in liquid culture at 40 °C.


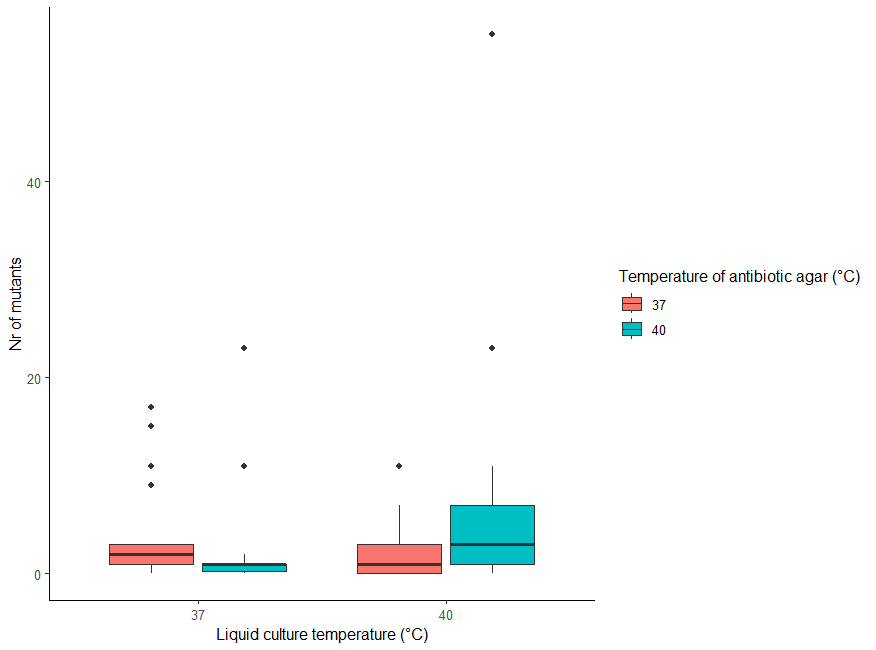


**Figure S12.**  Boxplot showing the number of ciprofloxacin-resistant mutants detected depending on the antibiotic agar incubation temperature. This plot shows the data populations grown in liquid culture at 37 °C (left) and at 40 °C (right).


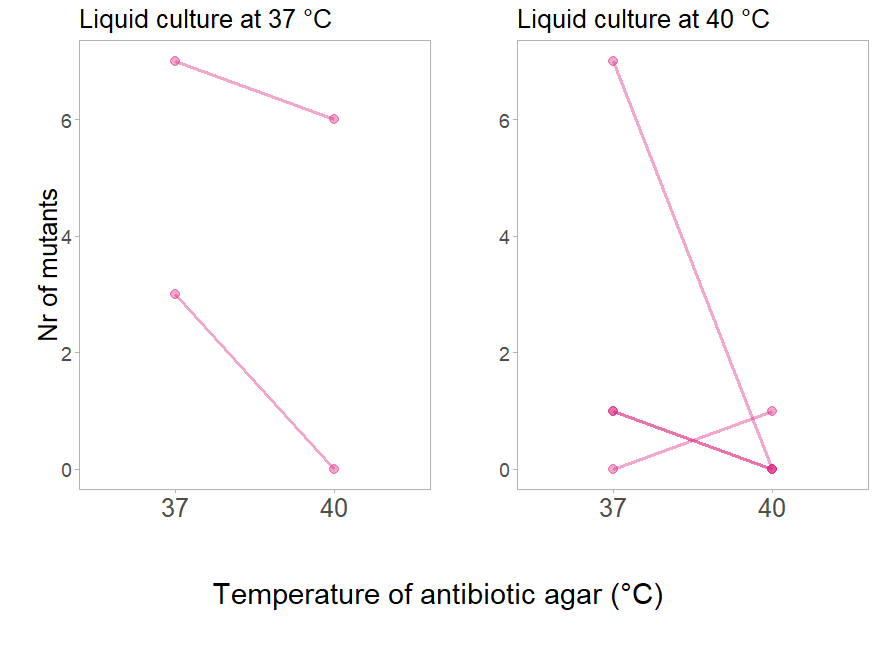


**Figure S13.**  The number of rifampicin-resistant mutants detected in each population at each antibiotic agar incubation temperature, lines connect samples originating from the same liquid culture. The left panel shows populations grown in liquid culture at 37 °C. The right panel shows populations grown in liquid culture at 40 °C.


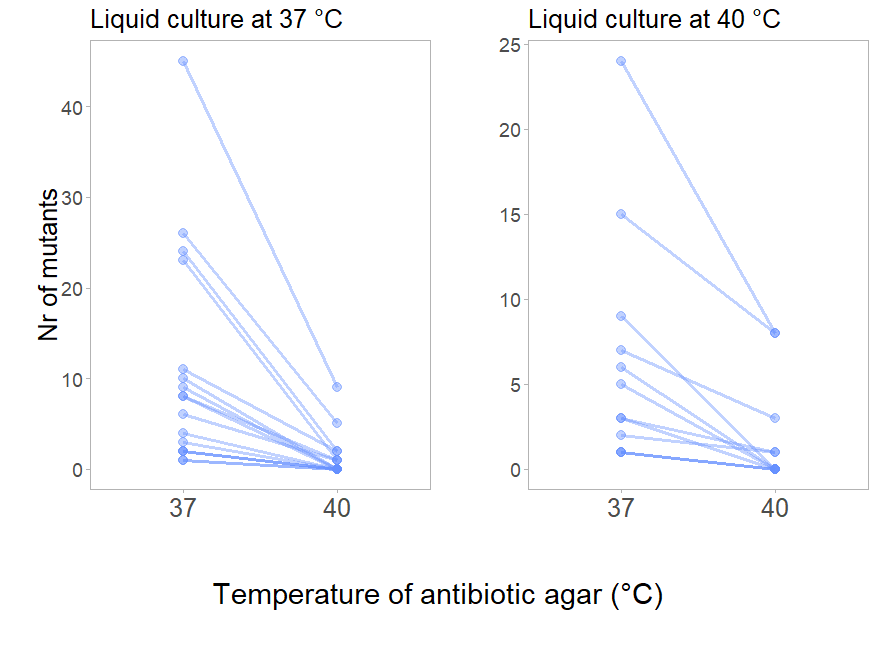


**Figure S14.**  The number of ampicillin resistant mutants detected in each population at each antibiotic agar incubation temperature, lines connect samples originating from the same liquid culture. The left panel shows populations grown in liquid culture at 37 °C. The right panel shows populations grown in liquid culture at 40 °C.


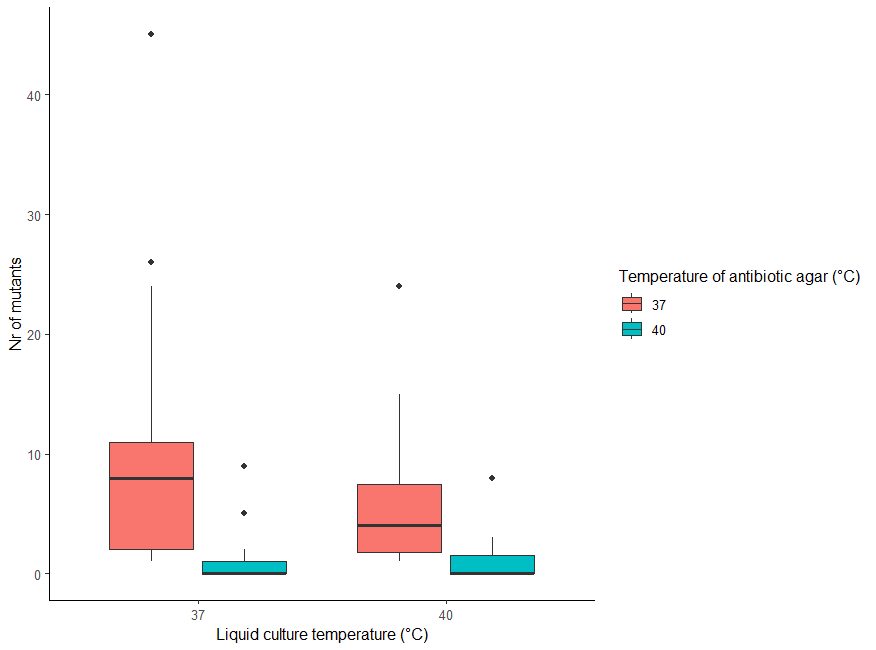


**Figure S15.**  Boxplot showing the number of ampicillin-resistant mutants detected depending on the antibiotic agar incubation temperature. Shown for populations grown in liquid culture at 37 °C (left) and at 40 °C (right).

# References

Deatherage, D.E., Barrick, J.E. (2014). Identification of mutations in laboratory-evolved microbes from next-generation sequencing data using *breseq*. *Methods in Molecular Biology,* 1151, 165–188.

Elena, S. F., Ekunwe, L., Hajela, N., Oden, S. A., & Lenski, R. E. (1998). Distribution of fitness effects caused by random insertion mutations in *Escherichia coli*. *Genetica*, *102*, 349-358.

Forst S., Delgado J., Inouye M. (1989) Phosphorylation of OmpR by the osmosensor EnvZ modulates expression of the ompF and ompC genes in *Escherichia coli*. *Proceedings of the National Academy of Sciences U S A.* 86(16), 6052-6056.

Gallet, R., Cooper, T. F., Elena, S. F., & Lenormand, T. (2012). Measuring selection coefficients below 10− 3: method, questions, and prospects. *Genetics*, 190(1), 175-186.

Howe, K., Karsi, A., Germon, P., Wills, R. W., Lawrence, M. L., & Bailey, R. H. (2010). Development of stable reporter system cloning luxCDABE genes into chromosome of *Salmonella enterica* serotypes using Tn7 transposon. *BMC microbiology*, 10(1), 1-8.

Jordan J.A., Lenski R.E., Card K.J. (2022) Idiosyncratic Fitness Costs of Ampicillin-Resistant Mutants Derived from a Long-Term Experiment with *Escherichia coli*. *Antibiotics* 11(3), 347.

Kishii R. & Takei M. (2009) Relationship between the expression of ompF and quinolone resistance in Escherichia coli. *Journal of Infection and Chemotherapy*. 2009 15(6):361-6.

Lenski, R. E., Rose, M. R., Simpson, S. C., & Tadler, S. C. (1991). Long-term experimental evolution in Escherichia coli. I. Adaptation and divergence during 2,000 generations. *The American Naturalist*, *138*(6), 1315-1341.

Lenski, R. E., & Travisano, M. (1994). Dynamics of adaptation and diversification: a 10,000-generation experiment with bacterial populations. *Proceedings of the National Academy of Sciences*, *91*(15), 6808-6814.

Li M., Liu Q., Teng Y., Ou L., Xi .Y, Chen S., Duan G. (2019) The resistance mechanism of Escherichia coli induced by ampicillin in laboratory. *Infection and Drug Resistance*, 12, 2853-2863.

Mazoyer, A., Drouilhet, R., Despréaux, S., & Ycart, B. (2016). flan: An R package for inference on mutation models. *The R Journal*, 2017, 9 (1), 334-351.

Nicoloff H., & Andersson DI. Lon protease inactivation, or translocation of the lon gene, potentiate bacterial evolution to antibiotic resistance. *Molecular Microbiology*, 90(6):1233-48.

Rodríguez-Verdugo, A., Gaut, B. S., & Tenaillon, O. (2013). Evolution of *Escherichia coli* rifampicin resistance in an antibiotic-free environment during thermal stress. *BMC evolutionary biology*, 13, 1-11.

Vinué L., Corcoran MA., Hooper D.C., Jacoby G.A. (2015) Mutations that enhance the ciprofloxacin resistance of *Escherichia coli* with qnrA1. *Antimicrobial Agents and Chemotherapy* 60(3), 1537-45.

Weinstein Z.B. & Zaman M.H. (2018) Evolution of rifampin resistance in *Escherichia coli* and *Mycobacterium smegmatis* due to substandard drugs. *Antimicrobial Agents and Chemotherapy* 63(1), e01243-18.
